# Investigation on the Factors Affecting *Cronobacter sakazakii* Contamination Levels in Reconstituted Powdered Infant Formula

**DOI:** 10.3389/fped.2015.00072

**Published:** 2015-08-24

**Authors:** Julio Parra-Flores, Alejandra Rodriguez, Francisca Riffo, Sofía M. Arvizu-Medrano, E. Verónica Arias-Rios, Juan Aguirre

**Affiliations:** ^1^Departamento de Nutrición y Salud Pública, Universidad del Bío-Bío, Chillán, Chile; ^2^Escuela de Medicina, Universidad de Concepción, Concepción, Chile; ^3^Departamento de Investigación y Posgrado en Alimentos, Facultad de Química, Universidad Autónoma de Querétaro, Querétaro, México; ^4^Department of Nutrition and Food Science, Texas A&M University, College Station, TX, USA; ^5^Department of Health Management, Atlantic Veterinary College, University of Prince Edward Island, Charlottetown, PE, Canada; ^6^Laboratorio de Microbiología y Probióticos, Instituto de Nutrición y Tecnología de los Alimentos (INTA), Universidad de Chile, Santiago, Chile

**Keywords:** *Cronobacter* spp, hypothetical dose, powdered infant formula, contamination levels, infants

## Abstract

**Introduction:**

Certain strains of *Cronobacter sakazakii* can cause serious invasive infections in children, mainly those <2 months old and fed with powdered infant formula (PIF). The infectious dose of *C. sakazakii* is unknown but evidence suggests that it is approximately 1000 colony forming units (CFU). PIF is currently considered safe if its end-product *C. sakazakii* level is <1 CFU/g. In this study, we determined the lag time, generation time (GT), and growth rate of five pooled *C. sakazakii* isolates to evaluate the factors affecting contamination levels in reconstituted PIF.

**Methods:**

1.71 log CFU/ml of *C. sakazakii* were inoculated into 100 and 3000 ml of reconstituted PIF and incubated at 22 and 35°C. Growth was evaluated over a 24-h period. ComBase was used for modeling.

**Results:**

In 3000 ml, the growth rate was 0.45 ± 0.02 log CFU/h with a lag phase of 3 ± 0.05 h and GT of 0.67 h at 22°C, while the growth rate was 0.73 ± 0.01 log CFU/h with a lag phase of 0.45 ± 0.03 h and GT of 0.41 h at 35° C.

**Conclusion:**

*Cronobacter sakazakii* grows rapidly in reconstituted PIF, especially at 35° C.

## Introduction

*Cronobacter* spp. are only rarely isolated from clinical specimens (1). Some species and strains have been isolated from normally sterile sites in hospitalized, elderly, and/or immunocompromised adults. These isolates were obtained while the patients were being treated for clinically symptomatic infections from more aggressive pathogens. *Cronobacter* were not causally linked to significant symptomatic illness in these patients (2). Other *Cronobacter* species, especially some strains of *Cronobacter sakazakii*, for example, strain type-4 (ST-4) or clonal complex 4 (CC4) (3), can infect healthy young infants and cause invasive and devastating illness (4). The most frequent clinical symptoms in reported cases of invasive pediatric *Cronobacter* infection are septicemia, necrotizing enteritis, and/or meningitis; the latter is associated with CC4 strains (3–5). Diarrhea and urinary tract infections have also been described (4–6). Reported mortality rates range from 42 to 80% for neonatal meningitis and 15 to 25% for septicemia (7).

The vast majority of invasive *Cronobacter* infections have been in infants <2 months old who were fed powdered infant formula (PIF). First reports of pediatric *Cronobacter* infections were in hospital outbreak settings. When PIFs were included as potential sources in outbreak investigations, contaminated PIF was repeatedly linked to the infections, microbiologically and epidemiologically (4). In 2002, health agencies recommended that PIF not be fed to hospitalized premature infants because of these findings. Since that time, most reported invasive *Cronobacter* cases have occurred sporadically in full-term infants living at home (4).

*Cronobacter* contamination has been repeatedly found in, and is sometimes endemic, in PIF and factories producing it. *Cronobacter* have also been repeatedly found in the ingredients used to make PIF and the factories processing these ingredients (8). PIF is not sterile and some of the organisms found in PIF can be pathogenic, including *Cronobacter* (9). Preparation utensils and equipment can become reservoirs of contamination when PIF is contaminated (10). When contamination occurs during production, it is not usually homogeneous throughout the contaminated production lot. Rather, the pattern depends on when and how microbial contamination occurred. For example, contamination from a colonized piece of equipment could be markedly sporadic. In general, *Cronobacter* contamination of PIF has been highly heterogeneous and is characterized by clustering and clumping, so that one portion of a contaminated product could have no detectable contamination even though another portion could contain concentrations above the infectious dose (11).

For some pathogens, contamination alone is enough to seriously jeopardize food safety. This is the case when the microorganism has a very low-infectious dose, that is, only a few live cells suffice to initiate the infectious process as in *Salmonella* and *Escherichia coli* O157:H7 (12–14).

The *Cronobacter* infectious dose is unknown and likely varies in relation to virulence factors and strain, as well as host species. Most research related to *Cronobacter* infectious dose was done before strain-typing schemes and assessment for virulence factors were feasible. A growing body of evidence supports the presence and role of various virulence factors in infantile *Cronobacter* infection (15–17). Pagotto et al. (18) intraperitoneally injected 10^7^ colony forming units (CFU) of one *Cronobacter* isolate into rats and provoked the disease and death. More recent research supports that the infectious dose for some *Cronobacter* isolates can be much lower. Iversen and Forsythe (19) proposed 1000 CFU as an approximate infectious dose. Research by Mittal et al. (17) and Richardson et al. (20) support this value.

Because the pediatric infectious dose for some pathogenic strains of *Cronobacter* is quite low, the initial contamination level of the microorganism and its distribution in PIF are extremely important factors in the risk associated with a contaminated PIF product (21). Another relevant factor is PIF water rehydration temperature. The FAO/WHO (22, 23) and WHO guidelines recommend using water at 70°C for PIF rehydration (24). Edelson-Mammel and Buchanan (25) estimated that using water at temperatures over 70°C to rehydrate PIF contaminated with *C. sakazakii* reduces 4 log CFU. Caubilla-Barron et al. (26) conclude that initial inactivation with rehydration water at 70°C controls any further growth that can occur in the cooled, reheated, and consumed formula (27).

In 2010, two cases of hemorrhagic diarrhea in hospitalized infants occurred in Mexico (6). Clinical isolates associated with these cases matched isolates recovered from unopened cans of PIF in the same hospital. The isolates were initially thought to be *Cronobacter*. This led to further investigation, as well as determining if the PIF was rehydrated according to the WHO home preparation guidelines, that is, 70°C. Water at this temperature kills *Cronobacter* cells (28). However, water used in the hospital was 45°C when mixed with PIF (6). Although healthcare workers attempted to follow these guidelines, this investigation determined that when PIF was mixed, water temperature had likely fallen to a level that would have incubated *Cronobacter* bacteria rather than killed it. It was also determined that caretakers outside the hospital setting frequently stored reconstituted PIF at room temperature for prolonged periods of time, even in geographic locations with high-ambient temperatures.

The objective of this study was to investigate the effect of temperature and volume in the growth of *C. sakazakii* in R-PIF. A secondary objective was to create awareness about this public health risk in developing countries where a lack of information is not only limited to the general public but also to health professionals.

## Materials and Methods

### Bacterial strains

Five strains of *C. sakazakii* were pooled as part of a microbiological, environmental, and clinical survey conducted between 2009 and 2011; all strains had been isolated in a Mexican hospital. To our knowledge, none of the five isolates were associated with any clinical symptomatology. Four of the strains were isolated from unopened cans of a single brand of PIF manufactured in Mexico. The other strain came from the interior of a feeding bottle that had been used to feed an infant. In the ongoing microbiological assessment, all bacteria suspected of being *Cronobacter* were initially evaluated using 16S rRNA amplification according to Lehner et al. (29) and rpoB gene (30). Based on multilocus sequence typing (MLST), the five strains described herein were later confirmed to be *C. sakazakii* clonal complex ST297 (31). This strain has not been associated with clinical disease in either infants or adults.

### Preparation of the bacterial suspension

The strains isolated from PIF were stored at 20°C in brain heart infusion (BHI) (Oxoid, Basingstoke, UK) with 20% glycerol. Strains were resuscitated in BHI at 35°C for 24 h in two successive passes to attain its maximum logarithmic growth phase. Each individual strain of *C. sakazakii* was separately evaluated in terms of PIF growth prior to its inoculation as a pool. Cells were harvested by centrifugation at 4500 rpm for 15 min at room temperature. Cell pellets were resuspended in peptone water 0.1% (Oxoid, Basingstoke, UK). Finally, the resulting suspension concentration was 10^9^ CFU/ml, which was confirmed by quantification in tryptic soy broth (TSB, Oxoid, Basingstoke, UK). Decimal dilutions were used for the inoculation of R-PIF and TSB.

To simplify the quantification of the pathogen in tryptic soy agar (TSA, Difco, Becton Dickinson, Sparks, MD, USA), *C. sakazakii* strains used in the test were resistant to 100 ppm Rifampin (Rif^+^). The Rif^+^ mutants were obtained from pure cultures by following the method published by Kaspar and Tamplin (32). Bacterial strains were cultured individually on TSA with Rifampin 100 ppm (Sanofi Aventi, Anagni, Italy) and incubated at 37°C for 24 h according to the methodology described by Neal et al. (33).

### Reconstitution and inoculation of R-PIF

Powdered infant formula was rehydrated (13% p/v) with 100 or 3000 ml sterile distilled water at 45°C. Each reconstituted formulas were inoculated with 1.71 log CFU/ml of the *Cronobacter* strain cocktail. The initial concentration was 1.71 log CFU/ml although the aim of the experiment was to reach 1.38 log CFU/ml, which was the concentration found in the refrigerated R-PIF reserve in the outbreak in Mexico (6). *C. sakazakii* ATCC 12868 and ATCC 29004 were grown in TSB and used as the control strains; uninoculated R-PIF was used as the negative control.

### Assessment of factors and definitions

*Cronobacte*r growth was studied using two temperatures (22 and 35°C) for two volumes (3000 and 100 ml). These temperatures were chosen based on the temperature of the milk kitchen and water bath (temperature used to warm R-PIF before feeding the infant). The volume was based on the quantity of PIF rehydration and average PIF consumption.

Three independent replicas of the growth curves were performed. Three samples from each replica were taken at 0, 1, 2, 3, 4, 6, 8, 12, and 24 h and plated on TSA supplemented with 100 ppm Rifampin. Plates were then incubated at 35°C for 24 h.

Generation time (GT) is the time required by the cells to duplicate. This parameter is estimated with the formula GT = log (2)/μ_max_. The growth rate (μ_max_) was calculated using DMFit version 2.0.

Maximum specific growth rate is the rate (log CFU per time point) where the population is divided during the exponential phase. The maximum is reached when the tangent of the curve during the exponential phase changes and starts to decrease. This is the kinetic parameter provided by DMFit when fitting the growth curves.

Lag phase is the period during which a cell or population adapts to the environmental conditions before it begins to duplicate.

### Statistical estimation of each factor’s effect on growth

Growth curves were fitted to the primary model of Baranyi and Roberts (34) to estimate the lag phase (λ) and the maximum specific growth rate (μ_max_) with the DMFit version 2.0 (Combase™) add-in for Excel 2007 Microsoft. An analysis of variance (SAS^®^9.4) was used to determine the statistical significance of the effects of temperature and volume on the growth rate.

## Results

### *Cronobacter* growth curves

**Figure 1:**
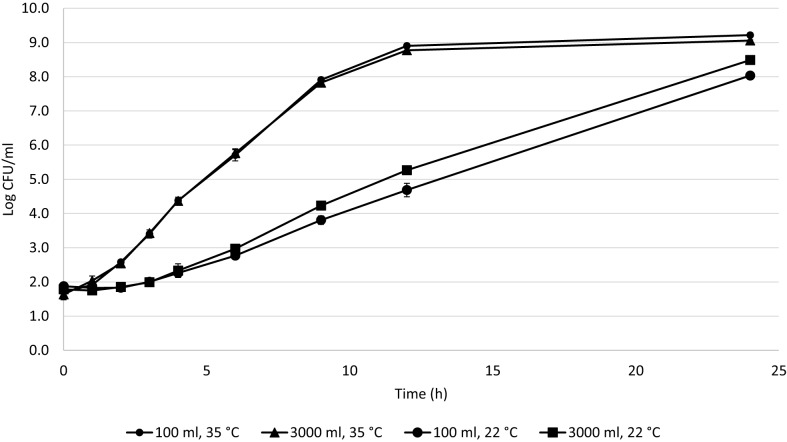
***Cronobacter* mean growth curves in 100 and 3000 ml at 35 and 22°C during 24 h**.

For each of the studied factors, triplicates were averaged and a good curve fit was obtained with DMFit Modeling (*R*^2^ = 0.99) (Figure 1).

The following parameters were determined from the fits. At 22°C in 3000 ml, the growth rate was 0.45 ± 0.02 log CFU/h with a lag phase of 3 ± 0.05 h and GT of 0.67 h. At 22°C in 100 ml, the growth rate was 0.32 ± 0.02 log CFU/h with a lag phase of 3 ± 0.02 h and GT of 0.94 h. At 35°C in 3000 ml, the growth rate was 0.73 ± 0.01 log CFU/h with a lag phase of 0.45 ± 0.03 h and GT of 0.41 h. At 35°C in 100 ml, the growth rate was 0.75 ± 0.02 log CFU/h with a lag phase of 0.53 ± 0.04 h and GT of 0.40 h. The growth rates were compared by Tukey’s test (SAS^®^, 9.4). The growth rate was affected by both temperature and volume (*p* = 0.0018); the factor with the most significant influence was temperature (*p* < 0.0001). When the interaction of these factors is evaluated, the growth rate is affected by volume at 22°C (*p* < 0.044), but is not affected at 35°C (*p* = 0.818).

### Estimated time for hypothetical infectious dose

**Figure 2:**
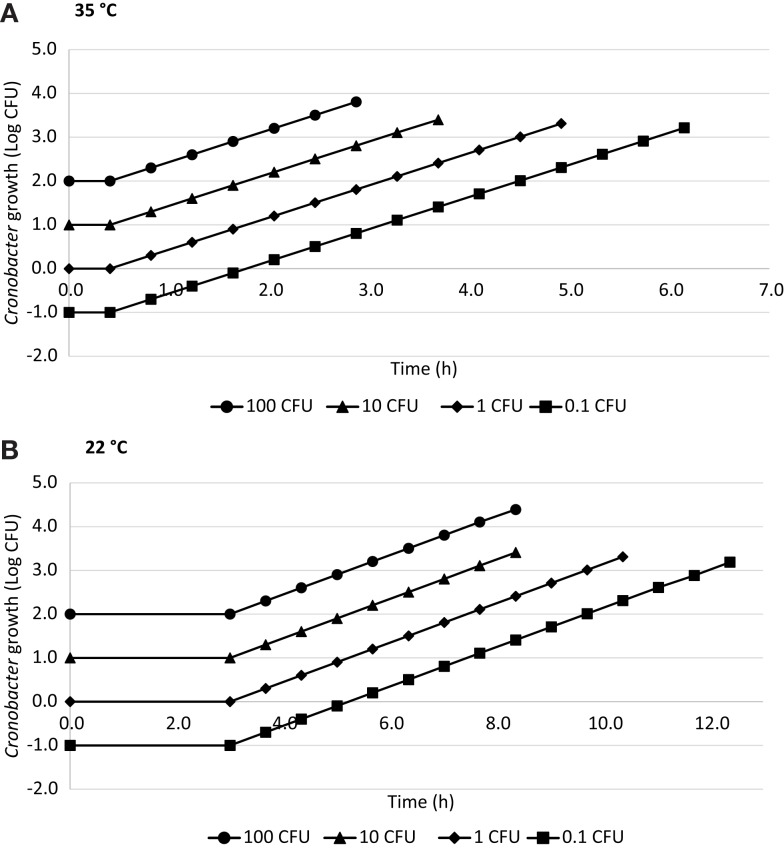
**Time to reach a hypothetical 1000 CFU dose in R-PIF at 35°C (A) and 22°C (B) with 0.1, 1, 10, and 100 CFU using ComBase generation time**.

The estimated time required to reach a hypothetical infectious dose of 1000 CFU (17, 19) calculated for 0.1, 1, 10, and 100 CFU of *Cronobacter* using ComBase™ is shown in Figure 2. The time needed to reach 1000 CFU at 35°C was 5.9 h when the initial concentration was 0.1 CFU, 4.5 h for 1 CFU, 3.2 h for 10 CFU, and 1.8 h for 100 CFU considering a lag phase of 0.45 h and GT of 0.41 h. At 22°C, these values were 11.9, 9.7, 7.5, and 5.2 h, respectively, with a lag phase of 3 h and GT of 0.67 h.

## Discussion

In literature, a fairly narrow range of lag and GTs have been reported for *Cronobacter*; however, the data on this topic are sparse and isolates were not characterized in terms of virulence factors or strain typing. For example, one study reported that three clinical and three PIF isolates had lag times between 1.8 and 3.4 h with a mean of 2.75 h and GT between 37 and 44 min when incubated at 23°C (26). The isolates and their sources were not characterized further and no data were provided for incubation at higher temperatures, for example, 35°C. In another study of nine isolates (three clinical samples, three environmental samples, and three PIF samples) in reconstituted PIF at 37°C, lag times were 2.2 to 3.0 h and GT for all isolates was 29 min (35). Kandhai examined one clinical and three PIF isolates and estimated a minimal lag time of 1.7 ± 0.44 h occurring between 37 and 39°C (36). Ghassem et al. (37) evaluated three *Cronobacter* strains isolated from R-PIF and one ATCC strain with ComBase and determined that GTs for four *Cronobacter* strains were 3.64, 0.50, 0.29, and 0.27 h and the lag phase in PIF was 10, 25, 37, and 45°C, respectively.

We examined the effects of two variables over time in relation to the *in vitro* growth of the pooled *C. sakazakii* isolates grown in reconstituted PIF: rehydration volume and incubation temperature. We did not vary reconstitution temperature or inoculum dose. At both evaluated temperatures, it is possible that rehydration volume did not significantly affect growth because the nutritional needs of our chosen inoculum were adequate at both volumes, however, additional research will be needed to confirm this hypothesis. This has practical implications for using PIF in countries where some caretakers store reconstituted formula at room temperature, especially in geographic areas with high-ambient temperatures. This result is also important when formula is stored appropriately and PIF is contaminated by virulent *C. sakazakii*, which could continue to grow in an infant’s gastrointestinal tract. An infant’s body temperature would be in the range of 36–40°C.

The WHO guidelines recommend that PIF be reconstituted with water at 70°C to inactivate any *Cronobacter* contaminating PIF (24, 28). However, there are at least three problems with this recommendation. First, some health agencies are concerned about the recommendation and do not support it (4). Second, it is unlikely that this recommendation will be followed in a home setting. Few caretakers will routinely measure the temperature of boiled water before they mix it with PIF. Third, rehydration instructions on some PIF labels do not comply with WHO guidelines. For example, in one Malaysian study, Abdullah Sani et al. found that six brands mentioned temperatures from 40 to 55°C, while all other brands indicated for previously boiled water to be “cool” or “lukewarm” to prepare PIF (38).

It is estimated that the infectious dose for *C. sakazakii* is low, approximately 1000 CFU (17, 19, 20); however, this is not based on the assessment of *Cronobacter* spp. virulent strains. Specifically, there are no data evaluating *C. sakazakii* ST-4 or CC4 strains, which have recently been reported as the major cause of *Cronobacter* meningitis in young infants (3, 16). Similarly, lag time, GT, and growth rate have not been determined for specific isolates associated with proven, invasive clinical infections. For these strains, all these parameters may well be lower than those reported in the literature, including in our own study.

In our study, we examined five apparently non-virulent strains of *C. sakazakii* that were isolated from PIF-related hospital-based sampling. We found that when we inoculated PIF at an initial level 1 CFU at 22°C, the generation or doubling time was approximately 1.0 h at 22°C and 0.5 h at 35°C. This is within the range of values found in previous studies, but not identical (26, 35–37). If we consider that the pathogen has been quantified in PIF with contamination levels of 0.22–1.61 MPN/100g (39), 0.23–1.91 MPN/100g (40), and 0.023–2.3 MPN/g (41), the possibility of illness associated with consumption at these contamination levels is very real. The heterogeneous distribution of *Cronobacter* contamination in PIF should be considered. A distribution of the inoculum size can be observed in a batch of food and this markedly influences public health risk. This heterogeneity can be due to the structural heterogeneity of the food matrix, incomplete mixing, incidental (post-processing) contamination, and/or localized microbial growth (42). It is critical to consider variability in microbial response because the credibility of a microbial risk assessment is based on its ability to consider the variability and uncertainty of each parameter involved in estimating final risk (43). The presence of a few atypical cells with short lag phase can unexpectedly shorten population lag time (44), which may shorten food shelf-life or, if pathogens are present in the food, increase the health risk to consumers.

This study has a number of limitations. First, the strains we tested were not associated with any known clinical illness. Second, using 1 CFU ignores the heterogeneous distribution of *C. sakazakii* contamination in PIF. In practical terms, when end-product testing results for a specific batch of product are below that level, an absence of high-level contamination in isolated portions of that batch is not guaranteed. Stratified random sampling enhances the probability of detecting heterogeneous contamination in dry PIF when compared to random sampling but will not necessarily detect isolated clusters of contamination (42). A third possible limitation of our study is that pooled strains do not accurately represent the individual strains in the pool. We examined a pool of five strains of *C. sakazaki* all associated with PIF. Before we pooled the isolates, we determined that the growth curves of the strains were similar and reached mean maximum values of 1.0 to 1.5 × 10^9^ (unpublished data). This was in part for convenience, but also because multiple strains have been involved in PIF outbreaks. Therefore, pooling isolates is more representative of the “real-world” situation. By pooling the strains, we introduced a naturally occurring uncertainty into our estimation.

In conclusion, 5.2 h at 22°C and 1.8 h at 35°C were needed to reach a hypothetical infectious dose of 1000 CFU (3 log) for the pooled isolates when starting with 100 CFU. The results of the present study support that *C. sakazakii* grows rapidly in PIF, especially at 35°C. There is an urgent need for ongoing training of personnel working in milk kitchens regarding hygiene practices, operational procedures, and the rigorous compliance of these measures. Furthermore, parents of infants consuming PIF must be informed about the risk of exposing their children when these are not immediately fed prepared R-PIF. The WHO guidelines regarding water rehydration temperature for R-PIF (>70°C) and feeding time should be promoted through education of health system users and professionals. Government should regulate PIF product labels and health authorities should strictly control and monitor PIF production. Whenever possible, infants <2 months of age should be only breast fed or fed ready-to-feed, pasteurized formulas because these are commercially sterile.

## Conflict of Interest Statement

The authors declare that the research was conducted in the absence of any commercial or financial relationships that could be construed as a potential conflict of interest.

## References

[1] Kucerova E Joseph S Forsythe S The *Cronobacter* genus: ubiquity and diversity. Qual Assur Saf Foods Crops (2011) 3:104–22. 10.1111/j.1757-837X.2011.00104.x

[2] Lai KK. *Enterobacter sakazakii* infections among neonates, infants, children, and adults. Case reports and a review of the literature. Medicine (2011) 80(2):113–22. 10.1097/00005792-200103000-00004 11307587

[3] Forsythe SJ Dickins B Jolley KA. *Cronobacter*, the emergent bacterial pathogen *Enterobacter sakazakii* comes of age; MLST and whole genome sequence analysis. BMC Genomics (2014) 15:1121. 10.1186/1471-2164-15-1121 25515150 PMC4377842

[4] Jason J. Prevention of invasive *Cronobacter* infections in young infants fed powdered infant formulas. Pediatrics (2012) 130:1–9. 10.1542/peds.2011-3855 23045556

[5] Bowen AB Braden CR. Invasive *Enterobacter sakazakii* disease in infants. Emerg Infect Dis (2006) 12:1185–9. 10.3201/eid1208.051509 16965695 PMC3291213

[6] Flores JP Arvizu Medrano S Silva Sánchez J Fernández-Escartín E. Two cases of hemorrhagic diarrhea caused by *Cronobacter sakazakii* in hospitalized nursing infants associated with the consumption of powdered infant formula. J Food Prot (2011) 74:2177–81. 10.4315/0362-028X.JFP-11-257 22186061

[7] Friedemann M. Epidemiology of invasive neonatal *Cronobacter* (*Enterobacter sakazakii)* infections. Eur J Clin Microbiol Infect Dis (2009) 28:1297–304. 10.1007/s10096-009-0779-4 19662446

[8] Reich F Konig R von Wiese W Klein G. Prevalence of *Cronobacter* spp. in a powdered infant formula processing environment. Int J Food Microbiol (2010) 140:214–7. 10.1016/j.ijfoodmicro.2010.03.031 20409601

[9] Beuchat L Kim H Gurtler J Lin L Ryu J Richards G. *Cronobacter sakazakii* in foods and factors affecting its survival, growth and inactivation. Int J Food Microbiol (2009) 136:204–13. 10.1016/j.ijfoodmicro.2009.02.029 19346021

[10] Bar-Oz B Preminger A Peleg O Block C Arad I *Enterobacter sakazakii* infection in the newborn. Acta Paediatr (1992) 90:356–8. 10.1111/j.1651-2227.2001.tb00319.x 11332182

[11] Jongenburger I Reij M Boer E Gorris L Zwietering M. Actual distribution of *Cronobacter* spp. in industrial batches of powdered infant formula and consequences for performance of sampling strategies. Int J Food Microbiol (2011) 151:62–9. 10.1016/j.ijfoodmicro.2011.08.003 21893361

[12] Hennessy TW Hedberg CW Slutsker L. A national outbreak of *Salmonella enteritidis* infection from ice cream. N Engl J Med (1996) 334:1281–6. 10.1056/NEJM199605163342001 8609944

[13] D’Aoust JY Arisand BJ Thisdale P *Salmonella eastbourne* outbreak associated with chocolate. Can Inst Food Sci Technol J (1975) 8:181–4. 10.1016/S0315-5463(75)73804-X

[14] Duncan SE Hackney CR Relevance of *Escherichia coli* O157:H7 to the dairy industry. Dairy Food Environ Sanit (1994) 14:656–60.

[15] Cruz A Xicohtencatl J Gonzalez B Bobadilla M Eslava C Rosas I. Virulence traits in *Cronobacter* species isolated from different sources. Can J Microbiol (2011) 57:735–44. 10.1139/w11-063 21859256

[16] Joseph S Forsythe S. Predominance of *Cronobacter sakazakii* sequence type 4 in neonatal infections. Emerg Infect Dis (2011) 17(9):1713–5. 10.3201/eid1709.110260 21888801 PMC3322087

[17] Mittal R Wang Y Hunter C Gonzalez-Gomez I Prasadarao N. Brain damage in newborn rat model of meningitis by *Enterobacter sakazakii*: a role for outer membrane protein A. Lab Invest (2009) 89(263–277):15. 10.1038/labinvest.2008.164 19139724 PMC3256236

[18] Pagotto FJ Nazarowec-White M Bidawid S Farber JM. *Enterobacter sakazakii*: infectivity and enterotoxin production in vitro and in vivo. J Food Prot (2003) 66:370–5. 12636287 10.4315/0362-028x-66.3.370

[19] Iversen C Forsythe S. Isolation of *Enterobacter sakazakii* and other *Enterobacteriaceae* from powdered infant formula milk and related products. Food Microbiol (2004) 21:771–6. 10.1016/j.fm.2004.01.00919729216

[20] Richardson A Lambert S Smith M. Neonatal mice as models for *Cronobacter sakazakii* infection in infants. J Food Prot (2009) 72:2363–7. 19903401 10.4315/0362-028x-72.11.2363

[21] Reij M Jongerburger I Gkogka E Gorris L Zwietering M. Perspective on the risk to infants in the Netherlands associated with *Cronobacter* spp. occurring in powdered infant formula. Int J Food Microbiol (2009) 36:232–7. 10.1016/j.ijfoodmicro.2009.07.011 19665815

[22] FAO/WHO. Enterobacter sakazakii and other Microorganisms in Powdered Infant Formula: Meeting Report. Microbiological Risk Assessment Series 6. Geneva: WHO/FAO (2004).

[23] FAO/WHO. Enterobacter sakazakii and Salmonella in Powdered Infant Formula: Meeting Report. Microbiological Risk Assessment Series 10. Geneva: WHO/FAO (2006).

[24] WHO (World Health Organization). Safe Preparation, Storage and Handling of Powdered Infant Formula: Guidelines. Geneva: WHO and the Food and Agriculture Organization of the United Nations (FAO) (2007).

[25] Edelson-Mammel S Buchanan R. Thermal inactivation of *Enterobacter sakazakii* in rehydrated infant formula. J Food Prot (2004) 67:60–3. 14717352 10.4315/0362-028x-67.1.60

[26] Nazarowec-White M Farber JM Incidence, survival and growth of *Enterobacter sakazakii* in infant formula. J Food Prot (1997) 60:226–30. 10.4315/0362-028X-60.3.226 31195476

[27] Caubilla-Barron J Kucerova E Loughlin M Forsythe S Bacteriocidal preparation of powdered infant formula FSA Project B13010 (2009). Available from: http://www.academia.edu/4018420/FSA_UK_Bacteriocidal_preparation_of_infant_formula

[28] WHO (World Health Organization). How to Prepare Formula for Bottle-Feeding at Home (2007). Available from: http://www.who.int/foodsafety/publications/micro/PIF_Bottle_en.pdf

[29] Lehner A Tasara T Stephan R. 16S rRNA gene based analysis of *Enterobacter sakazakii* strains from different sources and development of a PCR assay for identification. BMC Microbiol (2004) 4:43. 10.1186/1471-2180-4-43 15563736 PMC538263

[30] Stoop B Lenher A Iversen C Fanning S. Development and evaluation of rpoB based PCR systems to differentiate the six proposed species within the genus *Cronobacter*. Int J Food Microbiol (2009) 136:165–8. 10.1016/j.ijfoodmicro.2009.04.023 19467725

[31] Jackson EE Parra Flores J Fernandez Escartin E Forsythe SJ. Re-evaluation of a suspected *Cronobacter sakazakii* outbreak in Mexico. J Food Prot (2015) 78(6):1191–6. 10.4315/0362-028X.JFP-14-563 26038912

[32] Kaspar CW Tamplin ML. Effects of temperature and salinity on the survival of *Vibrio vulnificus* in seawater and shellfish. Appl Environ Microbiol (1993) 59(8):2425–9. 8368832 10.1128/aem.59.8.2425-2429.1993 PMC182301

[33] Neal J Marquez-Gonzalez M Cabrera-Diaz E Lucia L O’Bryan C Crandall P Comparison of multiple chemical sanitizers for reducing *Salmonella* and *Escherichia coli* O157:H7 on spinach (*Spinacia oleracea*) leaves. Food Res Int (2012) 45(2):1123–8. 10.1016/j.foodres.2011.04.011

[34] Baranyi J Roberts TA. A dynamic approach to predicting bacterial growth in food. Int J Food Microbiol (1994) 23:277–94. 10.1016/0168-1605(94)90157-0 7873331

[35] Lenati RF O’Connor DL Hebert KC Farber JM Pagotto FJ. Growth and survival of *Enterobacter sakazakii* in human breast milk with and without fortifiers as compared to powdered infant formula. Int J Food Microbiol (2008) 122(1–2):171–9. 10.1016/j.ijfoodmicro.2007.11.084 18207600

[36] Kandhai MC Reij MW Grognou C van Schothorst M Gorris LGM Zwietering MH. Effects of preculturing conditions on lag time and specific growth rate of *Enterobacter sakazakii* in reconstituted powdered infant formula. Appl Environ Microbiol (2006) 72:2721–9. 10.1128/AEM.72.4.2721-2729.2006 16597976 PMC1448981

[37] Ghassem M Babji AS Forsythe SJ Norrakiah AS Growth and survival of *Cronobacter* species as measured by media performance. Int Food Res J (2011) 18:367–72.

[38] Abdullah Sani N Hartantyo S Forsythe S. Microbiological assessment and evaluation of rehydration instructions on powdered infant formulas, follow-up formulas and infant foods in Malaysia. J Dairy Sci (2013) 96:1–8. 10.3168/jds.2012-5409 23141821

[39] Healy B Cooney S O’Brien S Iversen C Whyte P Nally J *Cronobacter* (*Enterobacter sakazakii*): an opportunistic foodborne pathogen. Foodborne Pathog Dis (2010) 7(4):339–50. 10.1089/fpd.2009.0379 19958103

[40] Siqueira-Santos RF da Silva N Junqueira V Kajsik M Forsythe S Pereira JL. Screening for *Cronobacter* species in powdered and reconstituted infant formulas and from equipment used in formula preparation in maternity hospitals. Ann Nutr Metab (2013) 63:62–8. 10.1159/000353137 23941974

[41] Parra J Oliveras L Rodriguez A Riffo F Jackson E Forsythe S Riesgo por *Cronobacter sakazakii* en leches en polvo para la nutrición de lactantes. Rev Chil Nutr (2015) 42(1):83–9. 10.4067/S0717-75182015000100011

[42] Jongenburger I Reij M Boer E Gorris L Zwietering M Random or systematic sampling to detect a localised microbial contamination within a batch of food. Food Control (2011) 22:1448–55. 10.1016/j.foodcont.2011.03.009

[43] Delignette-Muller ML Rosso L Biological variability and exposure assessment. Int J Food Microbiol (2000) 58:203–12. 10.1016/S0168-1605(00)00274-9 10939270

[44] Baranyi J. Stochastic modeling of bacterial lag phase. Int J Food Microbiol (2002) 73:203–6. 10.1016/S0168-1605(01)00650-X 11934028

